# Persistence of birth mode-dependent effects on gut microbiome composition, immune system stimulation and antimicrobial resistance during the first year of life

**DOI:** 10.1038/s43705-021-00003-5

**Published:** 2021-03-26

**Authors:** Susheel Bhanu Busi, Laura de Nies, Janine Habier, Linda Wampach, Joëlle V. Fritz, Anna Heintz-Buschart, Patrick May, Rashi Halder, Carine de Beaufort, Paul Wilmes

**Affiliations:** 1 Systems Ecology Group, Luxembourg Centre for Systems Biomedicine, University of Luxembourg, Esch-sur-Alzette, Luxembourg; 2 Translational Neuroscience group, Luxembourg Centre for Systems Biomedicine, University of Luxembourg, Esch-sur-Alzette, Luxembourg; 3 Metagenomics Support Unit, German Centre for Integrative Biodiversity Research (iDiv) Halle-Jena-Leipzig, Halle, Germany; 4 Department of Soil Ecology, Helmholtz-Centre for Environmental Research GmbH - UFZ, Halle, Germany; 5 Bioinformatics Core, Luxembourg Centre for Systems Biomedicine, University of Luxembourg, Esch-sur-Alzette, Luxembourg; 6 Centre Hospitalier de Luxembourg, Department of Pediatric Endocrinology and Diabetes, Luxembourg, Luxembourg; 7 Department of Pediatric Endocrinology, UZ Brussel, Vrije Universiteit Brussel, Brussels, Belgium; 8 Department of Life Sciences and Medicine, Faculty of Science, Technology and Medicine, University of Luxembourg, Esch-sur-Alzette, Luxembourg; 9 Present Address: Transversal Translational Medicine, Luxembourg Institute of Health (LIH), 1445 Strassen, Luxembourg

**Keywords:** Metagenomics, Microbiome, Antibiotics

## Abstract

Caesarean section delivery (CSD) disrupts mother-to-neonate transmission of specific microbial strains and functional repertoires as well as linked immune system priming. Here we investigate whether differences in microbiome composition and impacts on host physiology persist at 1 year of age. We perform high-resolution, quantitative metagenomic analyses of the gut microbiomes of infants born by vaginal delivery (VD) or by CSD, from immediately after birth through to 1 year of life. Several microbial populations show distinct enrichments in CSD-born infants at 1 year of age including strains of *Bacteroides caccae*, *Bifidobacterium bifidum* and *Ruminococcus gnavus*, whereas others are present at higher levels in the VD group including *Faecalibacterium prausnitizii*, *Bifidobacterium breve* and *Bifidobacterium kashiwanohense*. The stimulation of healthy donor-derived primary human immune cells with LPS isolated from neonatal stool samples results in higher levels of tumour necrosis factor alpha (TNF-α) in the case of CSD extracts over time, compared to extracts from VD infants for which no such changes were observed during the first year of life. Functional analyses of the VD metagenomes at 1 year of age demonstrate a significant increase in the biosynthesis of the natural antibiotics, carbapenem and phenazine. Concurrently, we find antimicrobial resistance (AMR) genes against several classes of antibiotics in both VD and CSD. The abundance of AMR genes against synthetic (including semi-synthetic) agents such as phenicol, pleuromutilin and diaminopyrimidine are increased in CSD children at day 5 after birth. In addition, we find that mobile genetic elements, including phages, encode AMR genes such as glycopeptide, diaminopyrimidine and multidrug resistance genes. Our results demonstrate persistent effects at 1 year of life resulting from birth mode-dependent differences in earliest gut microbiome colonisation.

## Introduction

The rate of caesarean section delivery is constantly increasing worldwide, which is partly driven by increases in overall income and access to health facilities.^1^ According to a 2015 report, 29.7 million births occurred via CSD in that year accounting for ~18% of the births in 169 countries.^1^ At 25% in Europe, this number is higher than the global average.^2^ The short-term risks of CSD include delayed or altered development of the immune system,^3^ reduced gut microbiome diversity,^4^ limited transmission of bacterial strains from mother to neonate^5,6^ and microbiome-borne functional deficiencies.^7–10^ Although few studies associate CSD with metabolic disorders^11,12^ and allergies,^13,14^ the long-term effects of birth mode are not well understood. Shao et al. reported that CSD may predispose individuals to colonisation by opportunistic pathogens including those carrying antimicrobial resistance (AMR) genes.^15^ On the one hand, several reports including our previously published study^8^ addressed questions concerning the very early development of the neonate’s gut microbiomes^14,16^ and immune system priming^3^ in relation to disease development.^17,18^ On the other hand, only few reports^19–22^ follow the effects of birth mode during the first year of life especially in relation to immune system priming, development and evolution of AMR, and the contribution of mobile genetic elements to the persistence of AMR genes.

Factors including environmental exposure,^14^ breast feeding and diet^19,23,24^ and genetics^25^ play crucial roles in the development of an infant. Aside from this, it is now generally accepted that birth mode, i.e. vaginal delivery (VD) or CSD, has a pronounced impact on early microbiome structure.^3,8,11,26,27^ While the majority of these studies focus on overall microbiome structure, analyses of the functional contribution of the microbiome has attracted attention due to its sensitivity to perturbation.^28^ For example, we previously reported that the microbiome in VD-born babies was enriched in bacterial genes encoding for lipopolysaccharide (LPS) biosynthesis, cationic antimicrobial peptide resistance as well as two-component systems.^8^ Interestingly, higher levels of LPS biosynthesis genes were associated with increased immune responses in VD neonates, whereas CSD neonates had reduced levels of TNF-α and IL-18 immediately after birth. Noteworthy in this context is previous work by Vatanen et al. which showed that differing LPS immunogenicity contributes to autoimmunity thereby affecting the long-term health outcomes of infants exposed to different antigens.^29^ Furthermore, others have hypothesized and reported^3,30^ a similar phenomenon, whereby the gut microbiome contributes to the development of the immune system during a “critical window” of development.^3,30–36^ In a neonatal cohort at risk for the development of asthma, bacterial metabolites were shown to specifically impede immune tolerance.^37^ However, some of the reports described above do not elaborate on the continuous effect of early immune system priming in the context of the birth mode and especially over the course of the first year of life, including whether these effects normalize over time.

Aside from the well-studied factors and consequences of development described above, the role of commensal microbiota in the emergence and spread of AMR is not well understood. Recent studies have reported that antibiotic exposure in infancy affects microbial diversity, and enriches AMR genes. Interestingly, Ravi et al. have suggested that the infant gut microbiome acts as a reservoir for multidrug resistance that persists throughout infancy up to 2 years of age.^38^ They reported that integrons (*int1* gene) in the gut could potentially be responsible for this phenomenon. Nevertheless, the effect of birth mode, CSD or VD, on the transmission and occurrence of AMR remains unresolved.

Here, we address the aforementioned gaps in knowledge concerning the effect of birth mode on the persistence of the gut microbiota over the first year of life including their inherent functions, immunogenic properties and their role in conferring AMR. Our results highlight birth mode-dependent differences in gut microbiome structure and their association with immune function. We found that the gut microbiota becomes similar between CSD and VD babies at 1 year of age, with the exception of an immunostimulatory commensal, *Faecalibacterium prausnitzii*, which was enriched in the VD group. In addition, we identified an increased abundance in AMR genes directed against synthetic and semi-synthetic antibiotics in CSD as early as 5 days *postpartum*. Strikingly, we found that mobile genetic elements (MGEs) including plasmids and bacteriophages are key contributors to the establishment and persistence of AMR, irrespective of birth mode. Collectively, our findings suggest that birth mode-dependent effects persist through the first year of life including the delayed immunostimulation of CSD infants likely affecting tolerance mechanisms as well as the apparent role of bacteriophages in conferring AMR.

## Results

### Birth mode-dependent gut microbiota differences during the first year

**Fig. 1:**
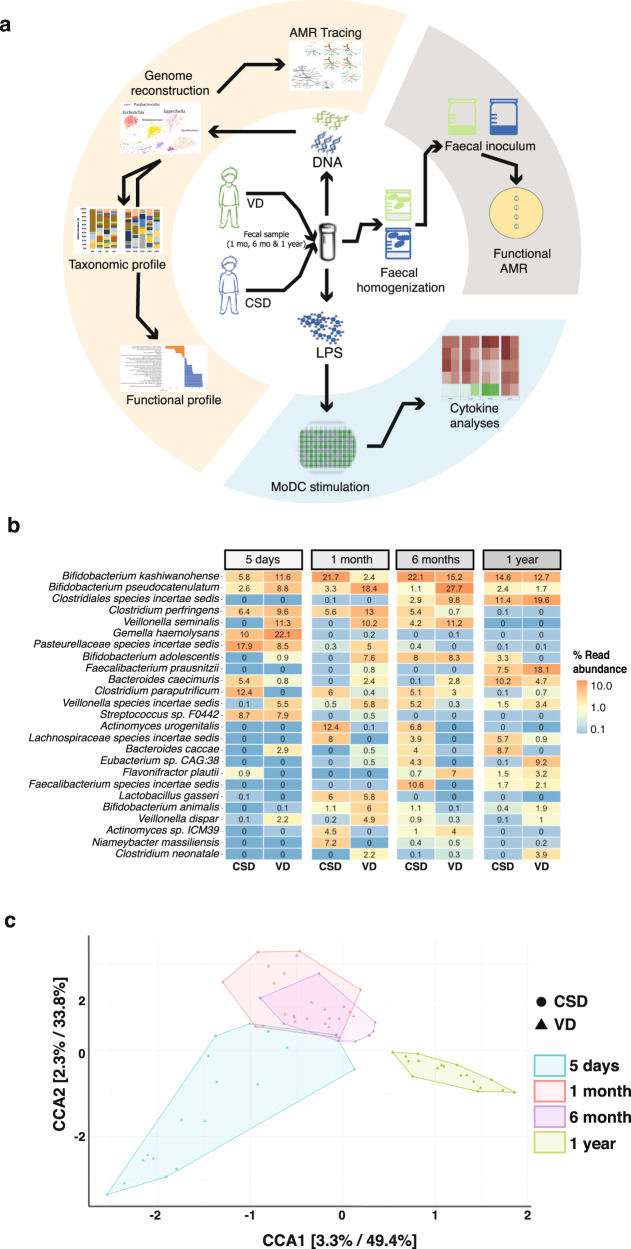
Gut microbiome profiles throughout the first year of life. **a** Workflow representation of DNA and LPS isolation from faecal samples for metagenomic, immune and functional AMR analyses. **b** Relative abundances of metagenomic operational taxonomic units (mOTUs) >1% abundance at day 5 after birth, 1 month, 6 months, and at 1 year of age. **c** Canonical correlation analyses (CCA) resolving the stratification of taxonomic profiles based on two covariates, i.e. birth mode and time when samples were sequenced.

We previously described the initial seeding and colonisation processes within the human gut microbiome and identified differences in microbiome structure and function as well as linked immunogenicity and immune system priming, which stratified according to birth mode.^8,39^ Building on this work, we aimed to understand the long-term effects in relation to the observed differences, especially through the first year of life which represents a “critical window” of development including physiological growth and immune system maturation. To achieve this, we followed VD and CSD neonates in our cohort and collected faecal samples at crucial intervals after birth, including 5 days, 1 month, 6 months and at 1 year of age (Fig. 1a). In one of our previous studies,^8^ a multivariate analysis was performed to compare the profiles of CSD (±SGA) to VD neonates. The results of these analyses demonstrated that delivery mode was the strongest determining factor in the microbial profile and predicted functions, irrespective of the infants were born SGA or not.^8^ In light of these analyses, we included the SGA samples within the CSD group. We reconstructed microbial genomes and identified differentially abundant taxa and functions between the groups using metagenomic sequencing data. Based on metagenomic operational taxonomic units (mOTUs), we calculated the Jensen–Shannon divergence index and found that the intra-group variability within CSD or VD was minimal while the inter-group variability between CSD and VD groups was significantly different (Supplementary Fig. 1). At the genus level, our data also recapitulated previously described^8^ significantly increased levels of *Bacteroides* (FDR-adjusted *p* < 0.05, Wilcoxon rank-sum test) in the VD neonates compared to CSD at the early timepoints (day 5 after birth and at 1 month). *B. caccae* also showed an increasing trend in the CSD group at 6 months and after 1 year of age, while *B. caecimuris* was significantly increased in CSD at 5 days after birth (Fig. 1b; FDR-adjusted *p* < 0.05, Wilcoxon rank-sum test) and showed an increasing trend at 1 year of age. However, at 1 year of age, the abundance of this genus in samples from CSD neonates was comparable to the levels in the VD group. In contrast, the levels of *Bifidobacterium* were increased in VD after 6 months, while *Faecalibacterium prausnitzii*, a commensal associated with healthy human microbiomes,^40^ was found to be significantly increased in the VD group at 1 year of age (Fig. 1b, FDR-adjusted *p* < 0.05, Wilcoxon rank-sum test). We further found that both birth mode and the neonatal age affect the gut microbiome community structure, whereby the latter contributes highly to variation within and between the groups (Fig. 1c). The taxonomic profiles at 1 year of age were distinct when compared to day 5, 1 month and 6 months from both groups.

### Assessment of differences in metagenomic functional potential at 1 year of age

**Fig. 2:**
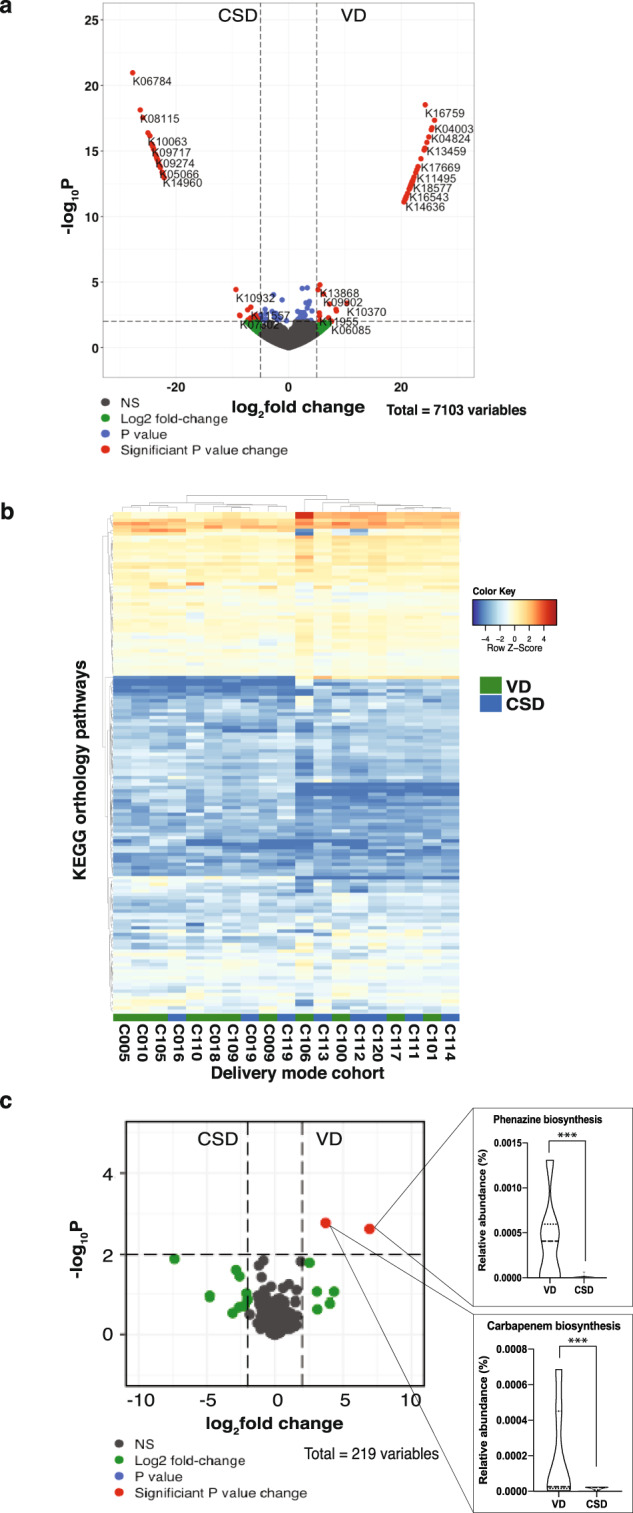
Functional differences at 1 year of age. **a** Volcano plot depicting the statistically significantly different KEGG orthologs found in both CSD and VD groups at 1 year of age. A total of 6413 variables were tested, with −log_10_(*p*-value) shown on the *y*-axis. Green dots indicate KOs with a fold change >2. Red dots indicate KOs with a significant fold-change cut-off of 2, and with a false-discovery rate-adjusted *p*-value cut-off of 0.01. **b** Heatmap based on the KEGG pathways found in both CSD and VD samples at 1 year of age. Each row denotes a pathway represented by the KO genes, with the hierarchical clustering being based on Euclidean distances using Ward’s clustering algorithm. **c** Volcano plot of the 219 KEGG pathways to which the KOs were mapped, tested for significance with a fold-change cut-off of 2, and with a false-discovery rate-adjusted *p*-value cut-off of 0.01. The insets show carbapenem and phenazine biosynthesis pathways that were statically significantly different. ***p-value < 0.001.

Taxonomic differences within the gut microbiome populations may not always manifest as differences in functional diversity due to the redundancy in the latter. To address this, we assigned KEGG^41^ orthology identifiers (KOs) to each gene identified from both groups. We found 84 differentially abundant KOs between VD and CSD samples at 1 year of age (Fig. 2a). In addition, we linked all identified KOs (*n* = 7103) to their corresponding KEGG orthology pathways (Fig. 2b) and performed differential pathway analyses. We found that the VD group showed an increase in the gene copy numbers of pathways involved in carbapenem and phenazine biosyntheses (Fig. 2c). We found that 21 unique genera were associated with carbapenem biosynthesis across both groups (Supplementary Data 1) spanning all major phyla found within the gut.

### Pro-inflammatory immune responses elevated in CSD after 1 year of life

**Fig. 3:**
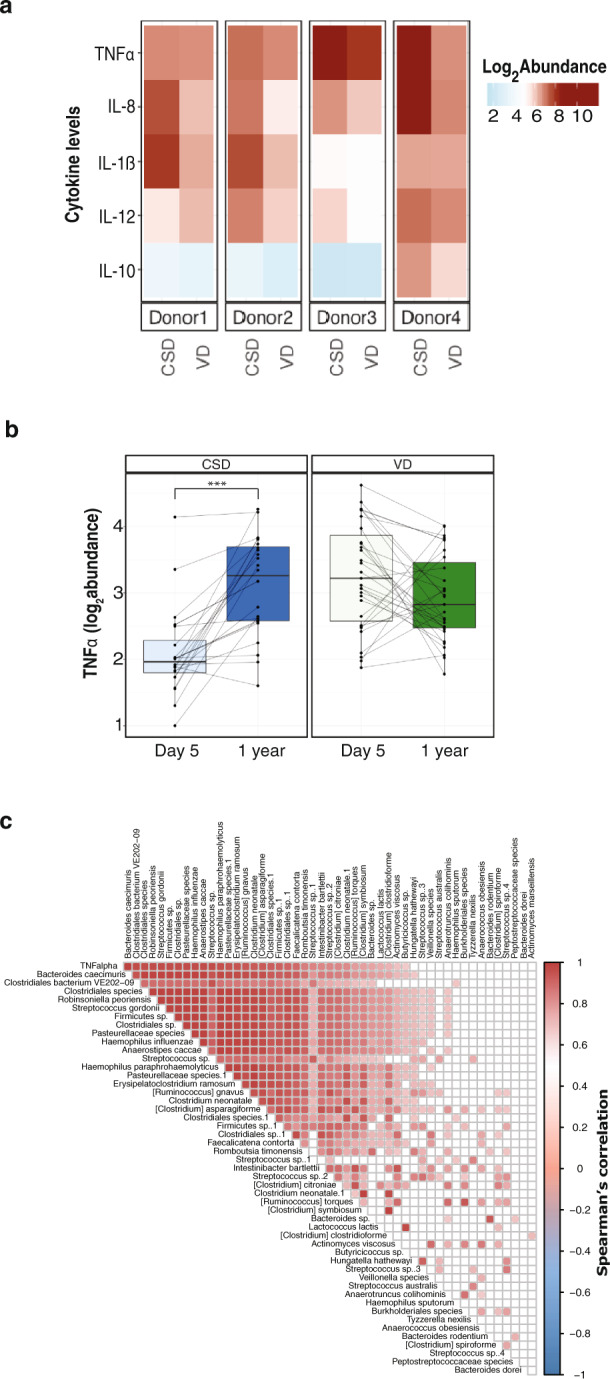
Immunostimulatory potential at 1 year of age. **a** Heatmap depicting the abundance (log_2_) of pro- and anti-inflammatory cytokines at 1 year of age. Cytokine levels were measured by stimulating MoDCs from healthy donors (Donor 1–4) with LPS isolated from faecal samples of CSD and VD neonates. **b** Boxplots depicting the TNF-α levels in both groups (CSD and VD) at day 5 after birth and 1 year of age. Paired two-way ANOVA (analysis of variance) *p*-values are listed in the plot to depict significant differences. ***p-value < 0.001. **c** Correlation of TNF-α levels (row 1) with the relative abundance of metagenomic OTUs based on canonical correlation analysis. Filled squares indicate significantly correlated taxa, whereas colour indicates positive (red) or negative (blue) correlation.

In the early stages of neonatal development, we found that the immune activation potential of LPS was significantly increased in samples from VD neonates,^8^ whereby the isolated LPS triggered the secretion of TNF-α and IL-18 by monocyte-derived dendritic cells (MoDCs) from four healthy adult donors. To determine if the immunostimulatory potential persisted at 1 year of age, we stimulated the MoDCs (obtained from four healthy adult donors) with LPS isolated from the faecal samples of the CSD and VD groups. In addition to TNF-α and IL-18, we also tested the potential of LPS to stimulate secretion of pro- and anti-inflammatory cytokines such as IL-1ß, IL-12, IL-8 and IL-10 (Fig. 3a). Interestingly, at 1 year of age, IL-18 was below the detection limits. We did not find any significant differences between the CSD and VD groups with respect to the levels of secreted TNF-α at 1 year of age. However, contrary to the patterns observed at 5 days after birth, we found that the levels of TNF-α stimulated by LPS were significantly increased at 1 year of age within the CSD group (*p* < 3.5 × 10^−5^, paired two-way ANOVA; Fig. 3b). Interestingly, the increase in stimulated TNF-α levels in CSD at 1 year of age was similar to the level of the cytokine stimulated by LPS from the day 5 VD samples (Fig. 3b). In addition, we found that the stimulated TNF-α levels at 1 year of age were positively correlated with the abundance of several mOTUs, including *Bacteroides caecimuris* and *Haemophilus influenzae* (Fig. 3c). Previous reports^42^ suggest that Enterobacteriaceae levels correlate with inflammatory levels. However, we did not find a correlation of these taxa with LPS levels in our study (Supplementary Fig. 2). Our data also indicate an increase in the number of Gram-negative (G-ve) bacteria at 1 year of age compared to day 5 after birth in the CSD group (Supplementary Fig. 3). The increase in Shannon diversity at 1 year of age compared to day 5 coupled with the increase in G-ve bacteria provides a mechanistic explanation why the LPS stimulation of donor cells from faecal samples of CSD resulted in similar levels of TNF-α (Fig. 3b), as observed with faecal samples from the VD group at 1 year of age.

### Antimicrobial resistance modulated by birth mode

**Fig. 4:**
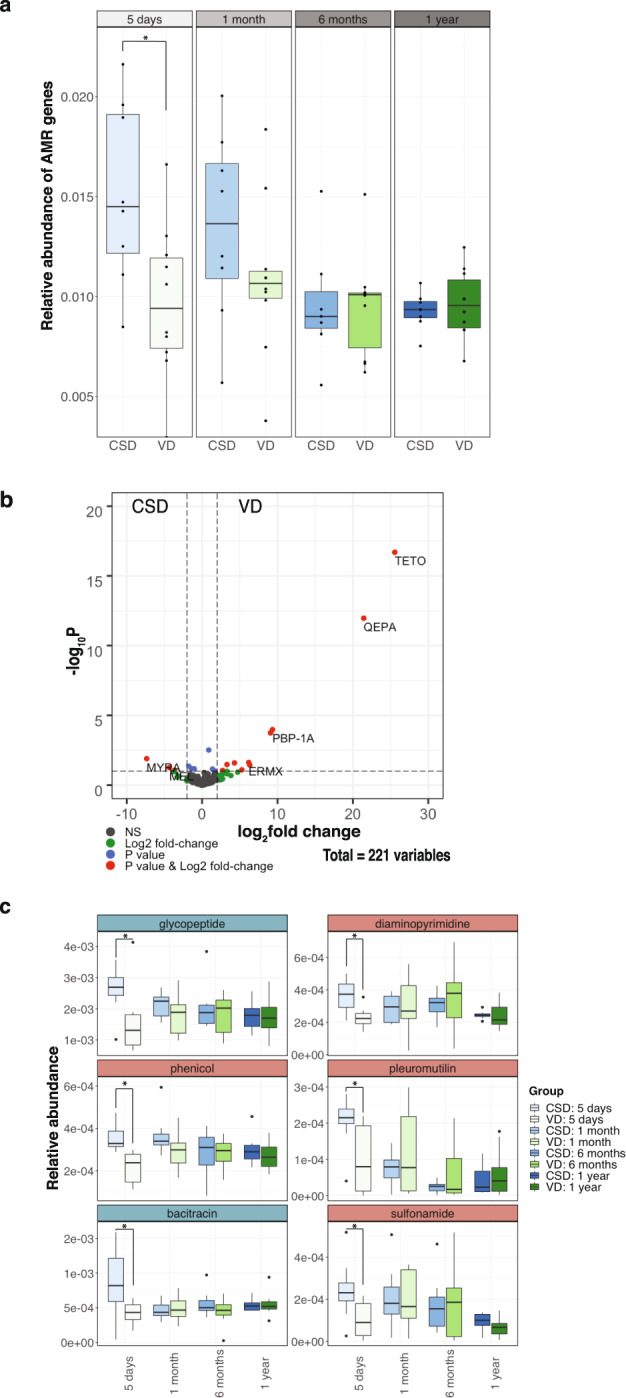
Antimicrobial resistance gene abundances over time. **a** Boxplots of the overall AMR gene abundance in CSD and VD samples at different timepoints including 5 days after birth and at 1 year of age. Wilcoxon rank-sum tests were used to test for significance. *p < 0.05. **b** Volcano plot depicting the significantly enriched genes in either CSD or VD samples at 1 year of age. **c** AMR categories which are significantly different between the groups at any of the timepoints are shown. Wilcoxon rank-sum tests were used to test for significance. *p < 0.05.

The analyses of the functional potential based on KEGG orthology revealed a stratification of antibiotic biosynthesis pathways based on whether an infant was born by CSD or VD (Fig. 2c). To assess and validate the impact of birth mode on the presence and persistence of AMR, we used a deep-learning approach^43^ to annotate antibiotic resistance genes in our metagenomic data.^44^ We determined the presence and relative abundance of AMR genes in samples collected from both CSD and VD at day 5 after birth, 1 month, 6 months and at 1 year of age. The samples collected from CSD neonates exhibited an increased abundance in AMR genes at the earliest time point (day 5) compared to the VD group. In addition, we found that the number of AMR genes detected in CSD infants at 1 year of age was significantly reduced in comparison to the CSD samples at day 5 (FDR-adjusted *p* < 0.0021, Wilcoxon rank-sum test; Fig. 4a, Supplementary Fig. 4a). To corroborate our observations on the levels of AMR genes, we assessed the abundance of AMR genes using a random subset of samples from the resistome study by Gasparrini et al.^45^ We found that the overall levels of AMR genes starting at 1 month through to 1 year of age were similar in their study to those observed in our own cohort (Supplementary Fig. 4b). Meanwhile in our study, at 1 year of age, we found several genes that were differentially abundant between the CSD and VD groups (FDR-adjusted *p* < 0.05, Wilcoxon rank-sum test; Fig. 4b). Since various genes can confer resistance to the same antibiotic, we regrouped the genes into their respective categories such as multidrug, tetracycline resistance etc. We found that genes conferring glycopeptide, phenicol, pleuromutilin, bacitracin, sulfonamide and diaminopyrimidine resistance were significantly increased in CSD compared to VD at day 5 after birth (Fig. 4c, Supplementary Fig. 5; FDR-adjusted *p* < 0.05, Wilcoxon rank-sum test). Interestingly, diaminopyrimidine, phenicol, pleuromutilin, and sulfonamide are synthetic or semi-synthetic antibiotics and, likely prevalent in the hospital environment.^46–49^ However, these differences did not persist over time. In addition, the mothers in our cohort across both groups (CSD:6 and VD:1) received prophylactic treatment against group B *Streptococcus* (Supplementary Data 2) in the form of cephalosporin. However, we did not find any distinguishing patterns within the resistance categories corresponding to this treatment regimen. Albeit a limited sample size, we also tracked the diet including feeding method (bottle- or breast-fed), antibiotic regimen and physical characteristics through the first year and did not find any significant correlations with functional pathways including AMR (Supplementary Fig. 6 and Supplementary Data 3).

### Taxa associated with antimicrobial resistance

**Fig. 5:**
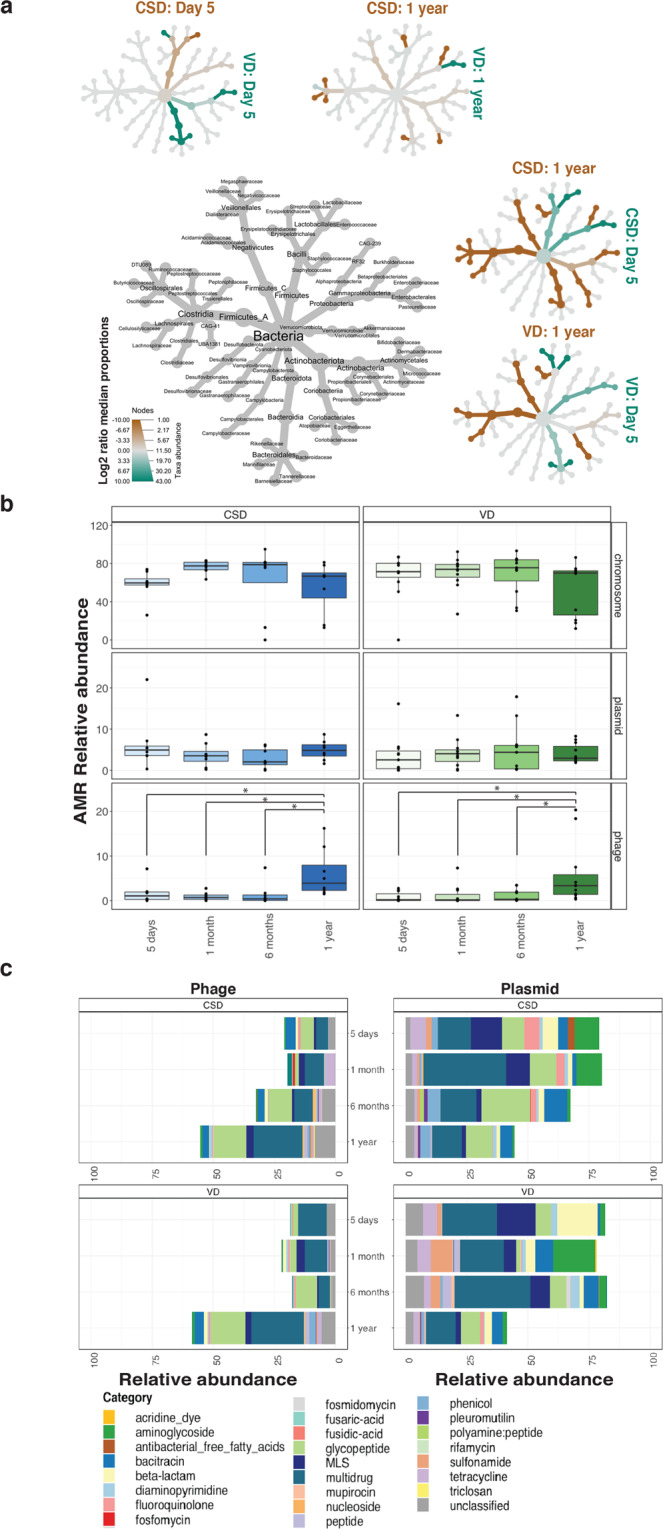
Taxa and mobile genetic elements associated with antimicrobial resistance. **a** Tree plots depicting the median proportion of AMR-associated taxa at day 5 after birth and at 1 year of age comparing the CSD and VD groups. Values used for plotting the trees are an average of all the samples within the group. **b** The relative abundances of AMR genes found on the bacterial chromosome, plasmids or phages at the different timepoints, ranging from day 5 after birth through to 1 year of age. CSD = blue (left panel), VD = green (right panel). Paired two-way ANOVA was used to assess significant differences; adjusted *p*-values are shown in the plot to depict significant differences. *p-value < 0.05. **c** Stacked bar plot depicting the AMR categories transmitted via phages and plasmids at all timepoints. Each colour in the plot is associated with a category listed in the legend on the right. The plot represents mean values for all samples in each group.

Since AMR genes were found in the metagenomic data, we investigated which taxa carried these genes by reconstructing metagenome-assembled genomes (MAGs), classifying them taxonomically based on the GTDB database,^50^ and linking AMR genes to individual MAGs. We then compared taxa contributing to AMR between birth mode at day 5 after birth and 1 year of age as well as the change over time within the individual groups (Fig. 5a).

Comparing CSD with VD, we found that shortly after birth, i.e. at day 5, *Bacilli* contributing to AMR were enriched in CSD, while *Bifidobacteriaceae*, *Bacteroidales*, *Eggerthellaceae*, and *Actinobacteria* linked to AMR were more abundant in VD samples. At 1 year of age, other taxa including *Burkholderiaceae*, *Ruminococcaceae* and *Bacteroidales* were prevalent in CSD samples (Fig. 5a). In contrast, taxa including *Enterobacteriales* were enriched in VD compared to CSD at 1 year of age.

We compared samples from day 5 after birth versus 1 year of age within each birth mode group independently to differentiate between taxonomic groups contributing to AMR. Within CSD samples, we found that *Enterobacteriales* and *Staphylococcaceae* were enriched at day 5 after birth while major AMR contributors at 1 year of age were *Lachnospiraceae*, *Bacteroidaceae*, *Actinobacteria* and *Oscillospirales*. Conversely, within VD samples early AMR resistance was mainly attributed to the abundance of *Bacteroidales*, *Lactobacillales*, *Propionibacteraceae* and *Enterobacteriaceae* at day 5 after birth. Meanwhile, at 1 year of age, VD samples were enriched in taxa including *Lachnospiraceae*, *Ruminococcaceae*, *Veillonellales* and *Eggerthellaceae* with respect to contribution of AMR genes to the resistome (Fig. 5a). These data suggest that AMR genes are also encoded by commensals apart from pathogens which, in the context of the present study, sustain their presence throughout the first year of life.

### Role of mobile genetic elements in antimicrobial resistance

Bacterial genomes have been fine-tuned over evolutionary timescales,^51^ potentially refining their defence mechanisms against various biocidal agents including chemicals. Aside from these, bacterial components such as MGEs are known to be potent factors in the spread of AMR^52^ and can transfer genes across distinct taxonomic clades. An example of such MGEs are plasmids as well as viruses including bacteriophages, which actively drive the transfer of genetic material.^53^ To determine the role of MGEs in conferring AMR in our neonate cohort, we analysed the genomic context of the AMR genes. The contigs were classified as chromosomal, plasmid, phage, ambiguous (those that could not be resolved) and unclassified. In this study, chromosomal sequences refer to the bacterial genome excluding plasmids, in accordance with the PlasFlow^54^ methodology. These criteria were used to assess the role of MGEs at all timepoints. The majority (average of ~75%) of the AMR genes were encoded on the bacterial chromosome (Fig. 5b). This phenomenon was prominent in the VD samples irrespective of sampling time point. On the other hand, the mean relative abundance of AMR genes encoded on plasmids (~5%) was marginally increased in the CSD group at both 5 days after birth and at 1 year of age. Overall, we found that phages encoded lower levels (1–3%) of AMR genes compared to the plasmids. However, we found that the relative abundances of phages encoding AMR were significantly increased after 1 year of age (Fig. 5b). Interestingly, we did not find any significant differences between the birth modes in relation to the virome profiles at any of the timepoints (FDR-adjusted *p* > 0.05, two-way ANOVA, Supplementary Fig. 7). However, a large proportion of the contigs were either ambiguous or unclassified but demonstrated an even distribution across all timepoints (Supplementary Fig. 8a). When ambiguous sequences mapping to both the bacterial chromosome and phages are included in the phage abundance metrics, it results in a higher abundance of AMR genes conferred by phage compared to plasmids (Supplementary Fig. 8b).

### Distribution of AMR categories encoded by mobile genetic elements

Assessing AMR conferred by MGEs, we found that both plasmids and phages encoded genes conferring resistance to several classes of antibiotics (Fig. 5c). Though significant differences were not apparent, we found that phage-encoded AMR genes against vancomycin (glycopeptide) and numerous other antimicrobials were dominant in both birth mode groups. In addition, plasmids conferred resistance to diaminopyrimidine and bacitracin, as well as β-lactams, phenicol, MLS and tetracyclines (Fig. 5c). Strikingly, these data suggest that MGE-mediated AMR, encoded by phages, is a potential factor in conferring AMR or serve as a reservoir for antimicrobial resistance throughout the first years of life.

### Phage-mediated horizontal gene transfer (HGT)

**Fig. 6:**
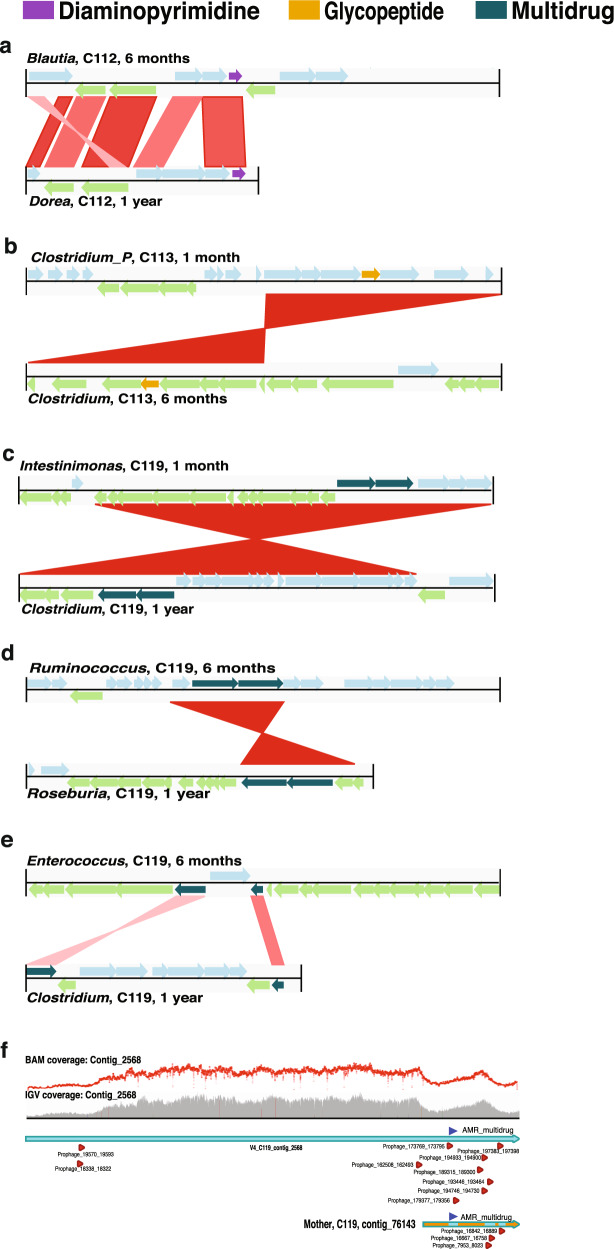
Horizontal gene transfer (HGT) events. Schematics depicting the **a** diaminopyrimidine resistance gene transfer between *Blautia* and *Dorea* in sample C112 (CSD). The lighter blue and green arrows represent genes localized on the forward and reverse strands, respectively. Red bars indicate matching regions between contigs with darker shades representing higher similarity. **b** HGT event transferring glycopeptide resistance between *Clostridium_P* and *Clostridium* in C113 (CSD). **c**–**e** Multidrug resistance genes were transferred horizontally between the following genera: *Intestimonas* and *Clostridium*, *Ruminococcus* and *Roseburia*, *Enterococcus* and *Clostridium*, in sample C119 (CSD). The timepoints at which the genomes were recovered and to which the HGT event corresponds are indicated next to the genera nomenclature. **f** Linear representation of ‘contig_2568’, which mapped to both bacterial chromosome and phage. Area plot (grey) and dot plot (red) indicating the coverage of the genomic regions. Triangles (red) indicate the prophage sequences (Supplementary Data 4) and their localisation coordinates along the contig, while the dark-blue triangle indicates the location of the multidrug AMR genes (*n* = 2) on the contig.

To understand phage-mediated horizontal gene transfer of AMR we analysed, in detail, phage contigs encoding AMR genes. We identified several genes that were horizontally transferred within the CSD and VD groups (Supplementary Data 4 and Supplementary Fig. 9). CSD samples (*n* = 3; C112, C113 and C119), exhibited HGT involving AMR genes including resistance to glycopeptide and multidrug (Fig. 6a–d). The majority (~88%) mapped to the bacterial chromosome. However, two genes encoding multidrug resistance were encoded by both chromosome and phage (C119: contig 2568, Supplementary Data 4). The contig was found to be a candidate prophage based on detailed inspection and was found to encode several genomic regions with prophage signatures flanking the multidrug resistance genes (Fig. 6f). In addition, the coverage of the contig across its entire length was more variable in the genomic regions where the prophage and AMR gene sequences were identified. Resolution of the taxa involved indicated HGT between the *Intestinimonas butyriciproducens* (GCA 003096335) and *Clostridium bolteae* (ATCC BAA 613 GCA 000154365) strains belonging to the *Oscillospirallales* and *Lachnospirallales* orders, respectively (Supplementary Fig. 10).

## Discussion

Birth mode is postulated to represent a major factor in shaping earliest gut microbiome colonisation and the linked development of neonates especially in relation to the priming of the neonates’ immune system.^3^ Apart from birth mode, additional aspects such as diet and medical factors have been described to have a significant effect on neonate colonisation and succession.^19^ Whether or not such effects persist during the first year of life remains an essential question. Here, we performed an in-depth longitudinal analysis of the gut microbiome using high-resolution metagenomics on samples collected during the first few days *postpartum* through to the first year of age. We specifically assessed the pervasive effect of birth mode-dependent microbiome differences in relation to immune system priming and AMR. In addition, we analysed the contribution of mobile genetic elements and the role of horizontal gene transfer in conferring AMR.

Our previous findings^8^ along with the longer-term trends of the present study underpin the notion that persistent structural and functional differences exist in the gut microbiomes of neonates born by CSD. More specifically, our results agree with other studies which have highlighted a reduced abundance and colonisation by taxa such as *Bifidobacterium* and *Bacteroides* in CSD neonates.^6,8,24,55,56^ In addition, we found that the levels of *Faecalibacterium prausnitzii* were significantly elevated in VD infants after 1 year of age. *F. prausnitzii* is a highly abundant commensal in the human gut including those with higher levels of diversity and richness when compared to individuals following a Western lifestyle.^57^ Concurrently, this taxon has been reported to be reduced in the gut of patients with ulcerative colitis and Crohn’s disease,^58^ which in turn may be linked to it being a keystone taxon conferring anti-inflammatory properties in humans.^59,60^ Further studies are necessary to effectively understand the longer-term consequences of the differential abundance of *F. prausnitzii* in humans beyond the first year of age.

Our previously published study^8^ highlighted a higher potential for LPS-mediated immune priming in VD compared to CSD at day 5 after birth. Conversely, we found that LPS extracts from the CSD samples taken at 1 year of age resulted in significantly higher TNF-α levels compared to 5 days after birth. Our results indicate that a reduction in the earliest immune system priming through key immunogenic molecules occurs in CSD neonates. This might lead to persistent effects throughout the first year of life which, in turn, may explain the higher rates of immune system-linked diseases observed in CSD infants in later life including metabolic disorders^11,55^ and allergies.^12,13^ Along these lines, Jakobsson et al. previously showed that children born via CSD have reduced Th1 responses.^4^ Furthermore, other groups have reported that early-life immune system stimulation impacts immune-disorders including asthma,^61^ allergies,^62^ diabetes and IBD.^63^ In this context, our findings indicate that birth mode-dependent gut microbiota alterations affect the status of the immune system throughout the first year of life, and likely beyond. This in turn may explain immunological deficits linked to numerous chronic diseases for which a higher propensity is observed in individuals born by CSD.^64^

Birth mode-associated alterations of the gut microbiota may facilitate colonisation by opportunistic pathogens, including those encoding antimicrobial resistance.^15^ Functional analyses of our metagenomic data highlighted enrichments in carbapenem and phenazine biosynthesis genes in the VD group after the first year of life, potentially a consequence of endogenous gut bacteria-mediated resistance mechanisms against opportunistic pathogens in the gut. Both carbapenem and phenazine are known to be bacterial compounds that are used clinically in fighting Gram-positive and Gram-negative pathogens.^65,66^ This data suggests that the indigenous gut microbiota plays a crucial, early role in conferring colonisation resistance against pathogens. In addition, we found that CSD is associated with resistance against semi- and synthetic antibiotics as early as 5 days after birth. It is well established that mothers undergoing CSD are administered antibiotics to prevent nosocomial infections, as a prophylactic policy.^42,67,68^ Interestingly, we did not find resistance towards the antibiotic treatment administered to mothers in our cohort. It, however, remains plausible that the enrichment in AMR genes especially against phenicol, pleuromutilin and diaminopyrimidine classes at day 5 after birth in CSD neonatal samples is linked to the hospital environment including the actual caesarean section.

In conjunction with the observed differences in AMR between CSD and VD our study also highlights the potential mode of AMR transmission via mobile genetic elements including via plasmids and/or bacteriophages.^69–71^ Parnanen et al. reported the presence of AMR genes and MGEs in infant faecal samples at 1 and 6 months of age.^72^ Our findings agree with their results and expand on these by additionally providing data on the abundance of AMR genes and MGEs at 5 days after birth. Furthermore, we identified 27 categories of AMR genes and linked these to both bacterial taxonomy and MGEs. We found that both plasmids and phages encoded genes which confer resistance to several classes of antibiotics. Of all MGEs, plasmids conferred resistance to a variety of antimicrobial compounds. Furthermore, we found that glycopeptide and multidrug resistance were transferred via phages, in accordance with previous reports.^73–75^ We also found that horizontal gene transfer plays a critical role in the continued transmission of AMR during the first year of life. While ~88% of HGT occurred via canonical methods involving the bacterial chromosome and plasmids, we found that prophages contributed to multidrug resistance in one CSD sample (C119). We detected prophage signature sequences flanking two multidrug resistance genes, horizontally transferred between bacteria from two distinct orders. Our findings thereby highlight the role of prophages, typically thought to mediate AMR in humans pathogens including *Staphylococcus aureus,*^76^ *Salmonella* and shiga toxin-producing *Escherichia coli*,^77^ as mediators of HGT even among commensals. Intriguingly, HGT events in the VD samples did not indicate any AMR gene transmission. On the other hand, considering the smaller sample size, further studies with an increased power are needed to clarify the role of phage-mediated AMR resistance especially during the first few days of life.

The persistence of differences in early-life exposure is an important but challenging research question, not least because of the paucity of long-term, longitudinal studies ranging from immediately after birth until early childhood. Our findings imply that birth mode leads to persistent gut microbiota structural and functional differences. We acknowledge that the limited sample size and the lack of detailed dietary information cannot rule out other confounding factors such as the in utero and postpartum environments of the infants. However, and importantly, our data suggest that gut microbiota structural and functional effects may predispose infants delivered by CSD to delayed immune priming resulting in a deficiency in tolerance. Our results pave the way for future, rational interventions aimed at restoring key functional features of the microbiota. In this context, further studies including following the children over extended periods of time are needed to understand birth mode-mediated manifestations of disease. Concurrently, an important research direction which arises from our study centres on the role of the gut mobilome in conferring AMR and how this affects microbiome trajectories and linked phenotypic outcomes in humans. Considering current global efforts directed at limiting the emergence of antibiotic resistance,^78^ appreciation of the role of phages as an additional source of resistance may be necessary for success in reducing the overall burden of AMR in the future.

## Methods

### Ethics statement

All aspects concerning the recruitment and collection of mother-neonate pairs including handling, processing and storing of samples as well as data were approved by the Luxembourg Comité national d’éthique de recherche, under reference number 201110/06 and by the Luxembourg National Commission for Data Protection under reference number A005335/R000058. Prior to specimen collection, following a detailed consultation; written and informed consent was obtained from all mothers enrolled in the study.

### Sample collection

Based on our previous study,^8^ the present study design aimed at testing the hypothesis that birth mode elicits longer-term functional microbiome changes which may impact neonatal health and development (with particular foci on antimicrobial resistance and lipopolysaccharide biosynthesis) and we performed the corresponding power analyses using data from our previous study. Founded on the increase in fold-change [caesarean section delivery (CSD) versus vaginal delivery (VD)] in antimicrobial resistance genes, a sample size calculation revealed a minimum number of four individual mother–infant pairs per group to achieve a power of 80% with a significance threshold of 5%. For the LPS-mediated functional cytokine measurements, we estimated a minimum sample size per group of six pairs based on a fold-change of 1.40*x* in TNF-α, i.e., a 40% difference of means between the samples (Supplementary Fig. 11). As previously published,^8,39^ we found that the functional microbiome differences provide clearer delineations when comparing groups than the typically reported taxonomic profiles. Based on our hypothesis, we focused on functional endpoints, in particular on the emergence and acquisition of AMR genes and the LPS-mediated immune stimulation. As per the results of the power analyses highlighting a minimum requirement of 6 mother–infant pairs per group, we further inflated the per-group sample size by 50% leading to a minimum of 9 mother–infant pairs per group. In the present study, babies delivered via caesarean (CSD, *n* = 11) and vaginal (VD, *n* = 9) deliveries were sampled during the first days of life and were followed-up at 1 month, 6 months and at 1 year of age (Supplementary Data 2). Samples were collected during follow-up visits into sterile plastic vials and immediately flash-frozen in liquid nitrogen. Faecal samples were stored until further processing at −80 °C.

### Faecal processing and nucleic acid extraction

Genomic DNA was isolated from 50 mg of frozen stool samples aseptically weighed into sterile vials, prior to processing with the DNeasy PowerSoil Kit (Qiagen, Luxembourg) including an additional incubation step at 65 °C and milling, as described previously.^8^ All the study samples yielded sufficient DNA for metagenomic sequencing including artefact-curated metagenomic data as described previously^8^ for subsequent analyses. DNA extracted from all timepoints was thereafter stored at −80 °C until further use.

### DNA sequencing

All DNA samples were subjected to random shotgun sequencing. Briefly, 250 ng of DNA was sheared using Bioruptor NGS (Diagenode, UCD300) with 30-s ON and 30-s OFF for 15 cycles. The sequencing libraries were prepared using TruSeq Nano DNA library preparation kit (Illumina, FC-121-4002) using the protocol provided with the kit. The libraries were prepared considering 350 bp average insert size. Prepared libraries were quantified using Qubit (Invitrogen) and the quality was checked on a Bioanalyzer (Agilient). Sequencing was performed on the NextSeq500 (Illumina) instrument using 2 × 150 bp read length at the LCSB Sequencing Platform.

### Data processing for metagenomics, including genome reconstruction

Paired forward and reverse sequences were processed using the metagenomic workflow of the Integrated Meta-omic Pipeline^79^ (IMP). The metagenomic processing workflow includes pre-processing, assembly, genome reconstruction and functional annotation of genes based on custom databases in reproducible manner. Briefly, the adapter sequences were trimmed in the pre-processing step including the removal of human reads. Thereafter the de novo assembly was performed using the MEGAHIT (version 2.0) assembler.^80^ Default IMP parameters were retained for all samples. Subsequently, we used MetaBAT2^81^ and MaxBin2^82^ for binning in addition to an in-house binning methodology previously described.^83^ This involved ignoring ribosomal RNA sequences in kmer profiles, clustering from VizBin embeddings,^84^ using density-based non-hierarchical clustering algorithms and depth of coverage for genome reconstructions. The reconstructed genomes are hereafter referred to as bins or metagenome-assembled genomes (MAGs). We obtained a non-redundant set of MAGs using DASTool^85^ with a score threshold of 0.7 for downstream analyses.

### Metagenomic taxonomic classification, virome and functional analyses

Trimmed and pre-processed read pairs were used as input to determine the microbial abundance and population genomic profiles based on the mOTUs^86^ (version 2) tool. Based on the marker genes in the mOTU2 database taxonomic profiling was performed. The relative abundances of the mOTUs were estimated using a minimum alignment length of 125 basepairs (bp), where the read counts were normalized to the gene length while also accounting for base coverage of the genes. This was done using the *motus profile* option with the built-in option (-c) for relative abundance values per samples. Simultaneously, to improve specificity and minimise false positives, a cut-off of seven genes that deviated from the median was used as an additional parameter to improve both sensitivity and precision. For the reconstructed MAGs, completeness and contamination were determined using CheckM,^87^ while the taxonomy for each MAG was assigned using the GTDB (Genome Taxonomy Database) toolkit (gtdb-tk)^50^ using the *lineage_wf* option and by using the fasta files as inputs for the MAGs.

For the analyses of functional potential from the assembled contigs, open-reading frames were predicted from the assembled contigs using a modified version of Prokka^88^ that includes Prodigal^89^ gene predictions for complete and incomplete open-reading frames. The identified genes were annotated with a hidden Markov models^90^ (HMM) approach, trained using an in-house database^84^ including all KO,^41^ TIGRFAM and SWISS-PROT^91^ groups and using *hmmsearch* from HMMER 3.1.^92^ Where multiple functional groups were assigned to genes, the best hits based on bit scores were selected. FeatureCounts^93^ was used to extract the number of reads per functional category, using the arguments -p and -O, thus yielding counts for each functional category. After the LPS-cytokine analysis, insufficient faecal sample for one of the CSD samples (C118) remained for metagenomic sequencing. Therefore, the sample was removed from subsequent metagenomic analyses. For the virome analyses, we used an iterative annotation method to recover microbial (bacterial and archaeal) viruses,^94^ and subsequently taxonomically annotated using a network-based classification protocol defined by Bolduc et al.^95^ Samples C109 was not included in the virome analyses due to viral contigs being below detection confidence thresholds.

### Identification of antimicrobial resistance genes and association with mobile genetic elements

We used a deep-learning approach, DeepARG,^43^ to predict and identify AMR genes within our metagenomic data. The output from Prokka, i.e. the translated fasta sequence files for all open-reading frames, was used as input for the AMR analyses. AMR genes were collapsed into categories based on the Comprehensive Antibiotic Resistance Database (CARD)^44^ and identified using DeepARG. Thereafter, the relative abundance of the AMR genes was calculated using the Rnum_Gi method described by Hu et al.^96^

Identified AMR genes and their categories were consecutively linked to associated bacterial taxonomy using the metagenomic bin classification. Furthermore, AMR genes were linked to predicted mobile genetic elements (MGEs; phages and plasmids) to identify probable transmission of AMR between taxa. For the identification of plasmids in the metagenomic data, PlasFlow^54^ was used with a threshold for filtering set to 0.7. Simultaneously, DeepVirFinder^97^ and VirSorter^98^ were used to identify phage sequences within the VD and CSD groups. Predictions from both these tools were subsequently merged to obtain a comprehensive catalogue of phage sequences. For the prediction of phage sequences, the DeepVirFinder thresholds for filtering were set at a *p*-value of < 0.05 and a score of 0.7, while for VirSorter the category 1 and 2 predictions were used for downstream analyses. To link both the MGEs and the taxonomy to the AMR genes, we mapped the genes to assembled contigs, followed by identifying the corresponding bins (MAGs) to which the contigs belonged. By considering all different predictions of MGEs, a final classification was made based on the genomic contexts of the AMR genes encoded on plasmids, phages or chromosomes, including classification of those that could not be resolved (ambiguous). Those AMR genes that could not be assigned to either the MGEs or bacterial chromosomes were further referred to as unclassified genomic signatures. Certain AMR genes were encoded on both the bacterial chromosome and phage genomes (Supplementary Data 4). In such cases, we recorded the encoded AMR gene as being ambiguous. The confirmation of AMR genes and their associated mode of transfer was performed manually alongside the mapping of identical 1 Kbp flanking regions, via the MetaCHIP analyses pipeline.^99^ Briefly, groups of genes among all input MAGs with maximum average identity were considered putative HGT genes. To validate the predicted candidates, a pairwise BLASTN was used to assess each pair of flanking regions of 10 Kbp. Visual representations of the genomic regions were extracted alongside the results for visual interpretation and inspection. Coverage of the genomic regions was additionally assessed through the IGV viewer using the bam file, and manually plotted based on per base coverage statistics for the latter.

### LPS isolation and in vitro immunostimulation for cytokine profiling

From the 1 year of age time point, 150 mg faecal samples were weighed aseptically and lipopolysaccharide (LPS) was extracted alongside an extraction blank to serve as a negative control. We also used an in-house pure culture of *E. coli*, from which extracted LPS was used as a positive control. To maximise yields, the samples were divided into triplicates, i.e. 50 mg per vial, prior to LPS extraction using the hot phenol-water protocol as previously described.^8^ After extractions the triplicates from each sample were pooled and quantified using an endotoxin-detection assay (Endolisa, #609033, Hyglos GmbH, Germany). All samples produced sufficient quantities of LPS. The purified LPS was used to stimulate monocyte-derived dendritic cells (MoDCs). Briefly, primary human monocytes were derived from blood samples from four healthy donors obtained through the Luxembourg Red Cross. The monocytes were further differentiated into MoDCs, in RPMI 1640 medium (ThermoFisher Scientific) supplemented with 10% foetal bovine serum (ThermoFisher Scientific), 20 ng ml^−1^ of granulocyte-macrophage colony-stimulating factor (Peprotech, London, UK), 20 ng ml^−1^ IL-4 (Peprotech) and 1% penicillin–streptomycin (Invitrogen). Subsequently, the immunostimulatory potential of the LPS fractions isolated from the 1 year of age faecal samples was determined. For this, MoDCs were treated with LPS extracts from VD and CSD samples. The amount of LPS from each sample that was used to stimulate the MoDCs was adjusted as described by Wampach et al.^8^ Briefly, the MoDCs were stimulated with 7.5 µl/well of LPS while a positive control was established using 15 EU/well LPS isolated from *E. coli*, and a negative control was set up by incubating MoDCs with 7.5 µl/well of the LPS extraction blank. For the in vitro stimulation, the amount of MoDCs was 1 × 10^5^ cells/well. Treatments were performed on cells from all the healthy donor-derived samples, and analysed for the presence of pro- and anti-inflammatory cytokines (TNF-α, IL-8, IL-18, IL-1β, IL-12 and IL-10) using both Human Instant and uncoated ELISA kits (ThermoFisher Scientific).

### Data analysis

All figures for the study including visualizations derived from the taxonomic, functional and cytokine profiling were created using version 3.6 of the R statistical software package.^100^ DESeq2^101^ and Wilcoxon rank-sum tests with FDR-adjustments for multiple testing were used to assess significant differences for the AMR and taxonomic analyses whereas a paired two-way ANOVA (analysis of variance) within the *nlme* package was used for identifying statistically significant differences in the cytokine profiles. Volcano plots were generated using the *EnhancedVolcano* package.^102^ Corrplots were generated using the *corrgram* package developed for R.^103^ The *metacoder*^104^ package was used to visualize the AMR-linked taxonomy in R.

## Supplementary information

Supplementary Legends

Supplementary figure 1

Supplementary figure 2

Supplementary figure 3

Supplementary figure 4

Supplementary figure 5

Supplementary figure 6

Supplementary figure 7

Supplementary figure 8

Supplementary figure 9

Supplementary figure 10

Supplementary figure

Supplementary Dtata 1

Supplementary Dtata 2

Supplementary Dtata 3

Supplementary Dtata 4

Supplementary Dtata 5

## Data Availability

The sequencing data and the MAGs generated during the current study are available from NCBI under bioproject accession number PRJNA595749. Supplementary Data 1 lists the taxonomic classifications of the carbapenem biosynthesis KEGG pathway identified within the MAGs. A reporting summary for this article is available as a Supplementary Information file (Supplementary Data 2). Supplementary Data 3 provides the clinical characteristics of all babies including details on diet, antibiotic and growth tracking. A description of the HGT events between reconstructed genomes and the manual validation of phage-conferred AMR is available in Supplementary Data 4, while the accession numbers for the Gasparrini et al.^45^ sequence data are listed in Supplementary Data 5. Legends for the supplementary figures are provided in the Supplementary material file. A description of the AMR and HGT analyses including pre-processing steps along with the scripts and config files can be found at GitLab: https://git-r3lab.uni.lu/susheel.busi/cosmic2.
