# Supplementary material for: Persistence of birth mode-dependent effects on gut microbiome composition, immune system stimulation and antimicrobial resistance during the first year of life
Source: ISME Commun. 2021 Mar 26;1:8. doi: 10.1038/s43705-021-00003-5 (PMC9723731; doi:10.1038/s43705-021-00003-5)
Supplement: Supplementary file 1 — Supplementary Legends [file 43705_2021_3_MOESM1_ESM.docx]

**­Persistence of birth mode-dependent effects on gut microbiome composition, immune system stimulation and antimicrobial resistance during the first year of life**

***Supplementary Legends***

Supplementary Figure 1. Intra- and inter-birth mode variability

Intra- and inter-birth mode Jensen-Shannon divergences of the mOTU profiles were calculated and tested to assess intra- and inter-group variability. Statistically significant differences were determined using the Wilcoxon-Mann-Whitney test; ****p*<0.001.

Supplementary Figure 2. TNF-α correlation with Enterobacteriaceae

Log_2_ abundance of TNF-α levels were tested for correlation with the percent relative abundance of Enterobacteriaceae based on read counts mapping to the family. r^2^ indicates the Spearman correlation coefficient.

Supplementary Figure 3. Overall AMR abundance at early and at the one year timepoint

**a.** Barplots depicting the differential AMR abundance between CSD and VD groups comparing the metagenomic from day five after birth with one year of age. **b.** Time series analyses of all AMR categories within CSD and VD groups, spanning each timepoint between day five through to one year of age. The Y-axis depicts the relative abundance of the genes collapsed to AMR categories.

Supplementary Figure 4. Validation of AMR abundance levels

Overall AMR relative abundances observed in samples from the Gasparrini *et al.*^103^ study ranging from one month through to one year of age. Data and sample accession numbers are listed in Supplementary Data 3.

Supplementary Figure 5. Functional pathways from day 5 after birth through to one year of age

Heatmap showing the relative abundances of the functional (KEGG) pathways through the first year of life. The plot includes annotations for different categories including group, timepoint and diet, i.e. breast-milk, formula, mixed or no data available.

Supplementary Figure 6. Longitudinal virome profiles

Bubble plots showing the log relative abundance of viruses identified within the CSD and VD samples through the first year of life. Only those with relative abundance greater than 0.01% are depicted. Significance was tested using a Two-way ANOVA, for a FDR-adjusted *p* < 0.05.

Supplementary Figure 7. Abundances of bacterial chromosome and MGEs across time

**a.** Barplots depicting the relative abundance of ambiguous and unclassified sequences with respect to chromosomal or MGE classification. **b.** Distribution of chromosomal and MGE classification of AMR sequences at different time points for CSD and VD. Sequences assigned to both chromosome and bacteriophages were classified as belonging to bacteriophage under the assumption that the former represent likely prophages. **c.** The association of bacteriophage and plasmids with resistance categories at all timepoints comparing CSD and VD groups.

Supplementary Figure 8. Abundance of AMR and prophage signature genes

**a.** Barplots depicting the abundance of contigs with AMR and prophage signature genes along the y-axis. The x-axis shows the longitudinal abundance of CSD and VD samples. **b.** Relative abundance of AMR and prophage signature genes in CSD and VD samples from day five after birth through to one year of age.

Supplementary Figure 9. Strain resolution of recovered genomes

**a.** Neighbour-joining tree of the *Intestinimonas* genome involved in HGT recovered from the one month faecal sample of CSD sample C119. The tree was generated using ribosomal proteins in comparison to 1925 complete genomes obtained from the RefSeq database and visualised using the AnnoTree webserver^104^. **b.** Taxa identified as belonging to the genus *Clostridium* were mapped against the RefSeq database and identified using the above approach.

Supplementary Figure 10. Power analysis and sample-size estimation

**a.** Power analysis based on the increase in fold-change [caesarean section delivery (CSD) versus vaginal delivery (VD)] in antimicrobial resistance genes. Sample size calculation using our study design revealed a minimum number of four individual mother-infant pairs per group to achieve a power of 80% with a significance threshold of 5%. **b.** Sample size estimation based on the LPS-mediated cytokine measurements. An estimated minimum sample size per group of six pairs (Figure 1B), based on a fold-change of 1.40x in TNF-α, i.e. a 40% difference of means between the samples was calculated to be the requirement.

Supplementary Figure 11. Alpha diversity and Gram staining profiles

**a.** Shannon and Simpson diversity indices based on the mOTU counts measured through the first year of life. Significance was tested using a two-way ANOVA, for a FDR-adjusted *p* < 0.05. **b.** The number of Gram -ve and +ve organisms in CSD and VD. **c.** Volcano plots of the abundance of KEGG groups involved in LPS biosynthesis at day 5 of age. K12985: LPS 1,2-glucosyltransferase and K18827: O-antigen chain-terminating methyltransferase. **d.** Volcano plots of the abundance of KEGG groups involved in LPS biosynthesis at 1 year of age.
