# Supplementary figures and images for: Persistence of birth mode-dependent effects on gut microbiome composition, immune system stimulation and antimicrobial resistance during the first year of life
Source: ISME Commun. 2021 Mar 26;1:8. doi: 10.1038/s43705-021-00003-5 (PMC9723731; doi:10.1038/s43705-021-00003-5)

Supplementary Figure. 1

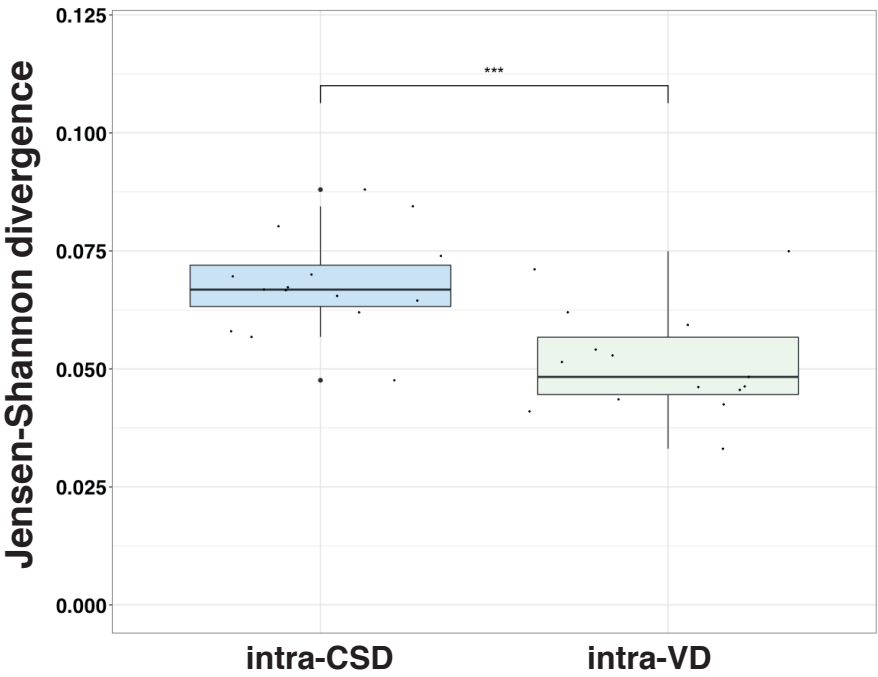

Supplement: Supplementary file 2 — Supplementary figure 1 [file 43705_2021_3_MOESM2_ESM.pdf]

Supplementary Figure. 2

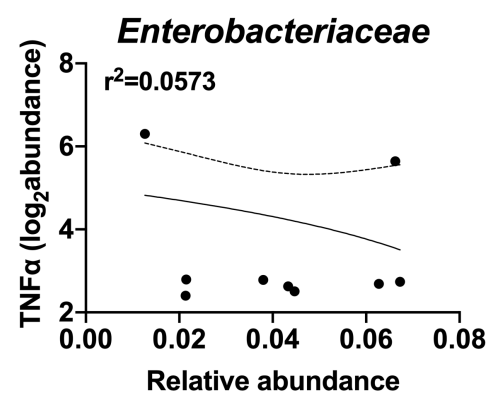

Supplement: Supplementary file 3 — Supplementary figure 2 [file 43705_2021_3_MOESM3_ESM.pdf]

Supplementary Figure. 3

**a**

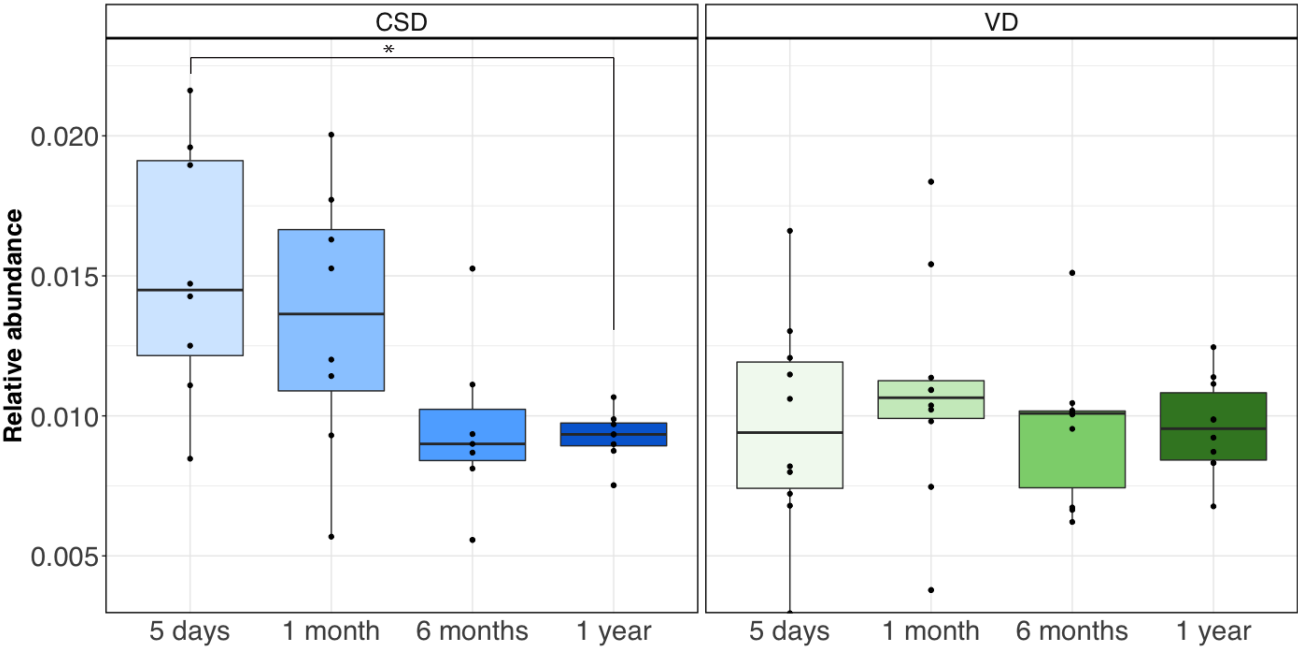

Supplementary Figure 3  
b (i)

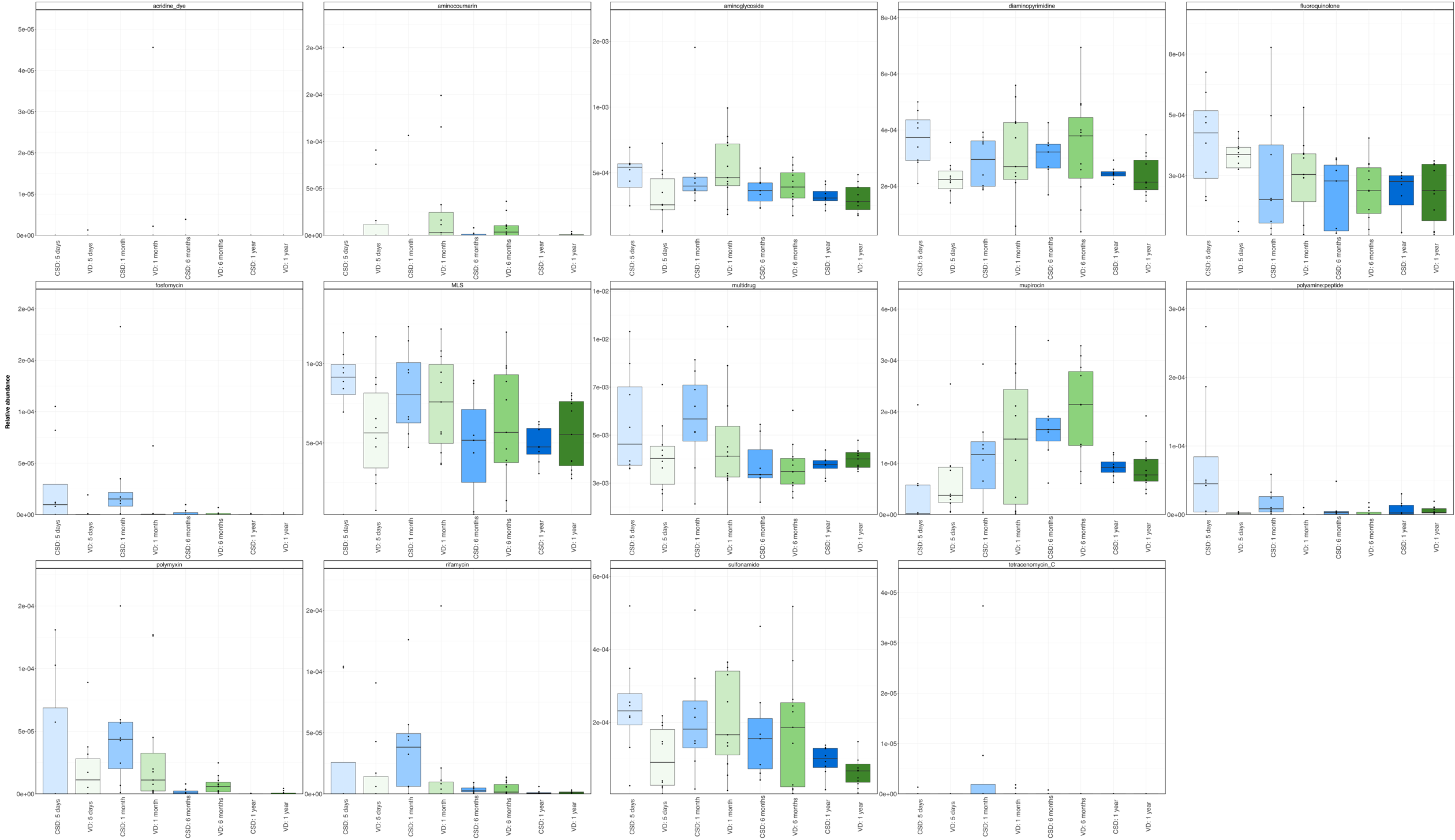

Supplementary Figure 3  
b (ii)

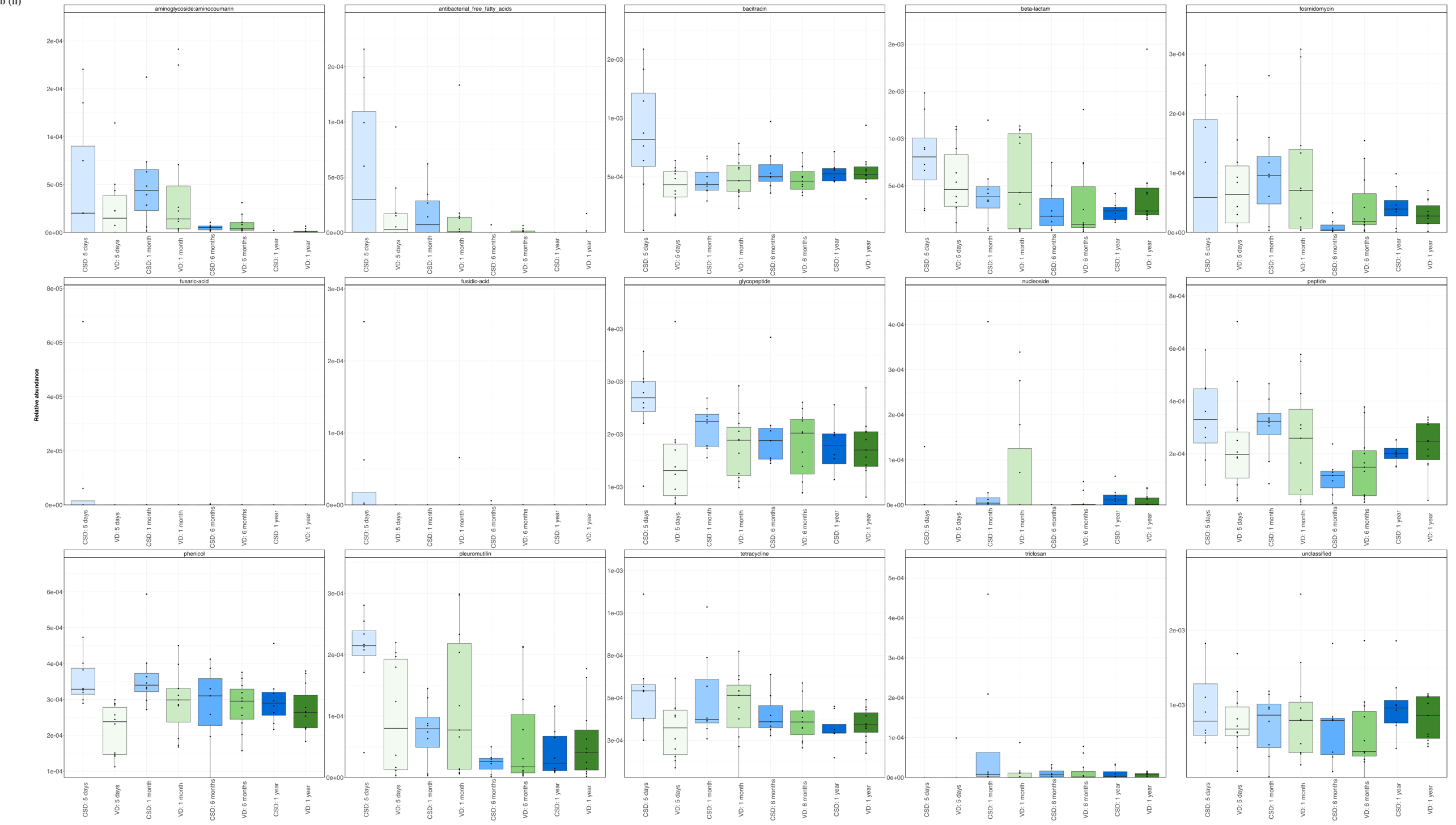

Supplement: Supplementary file 4 — Supplementary figure 3 [file 43705_2021_3_MOESM4_ESM.pdf]

Supplementary Figure. 4

a

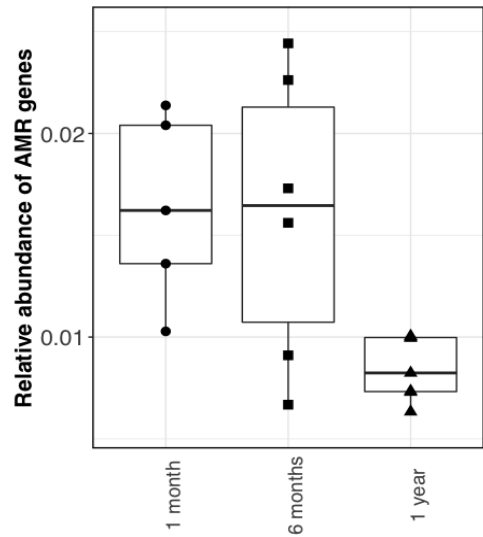

b

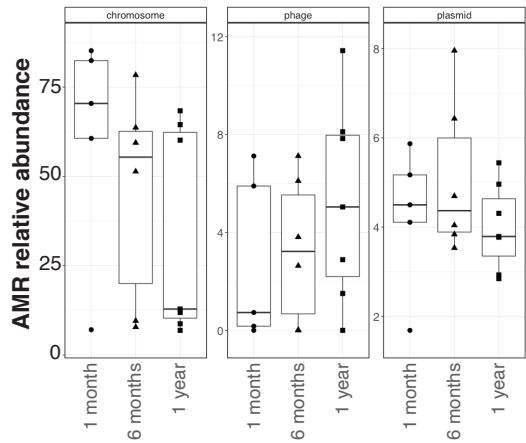

Supplement: Supplementary file 5 — Supplementary figure 4 [file 43705_2021_3_MOESM5_ESM.pdf]

Supplementary Figure. 5

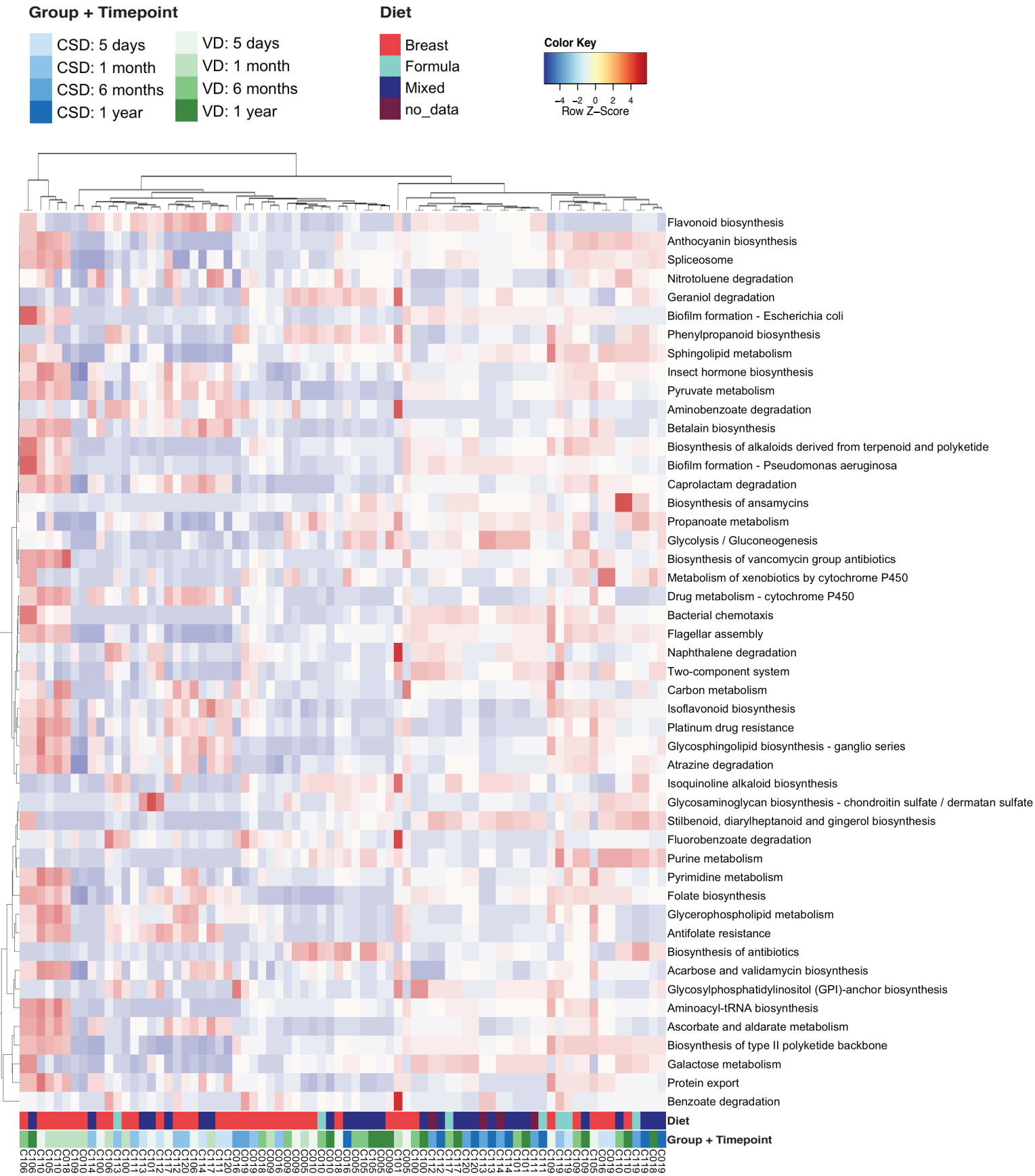

Supplement: Supplementary file 6 — Supplementary figure 5 [file 43705_2021_3_MOESM6_ESM.pdf]

Supplementary Figure. 6

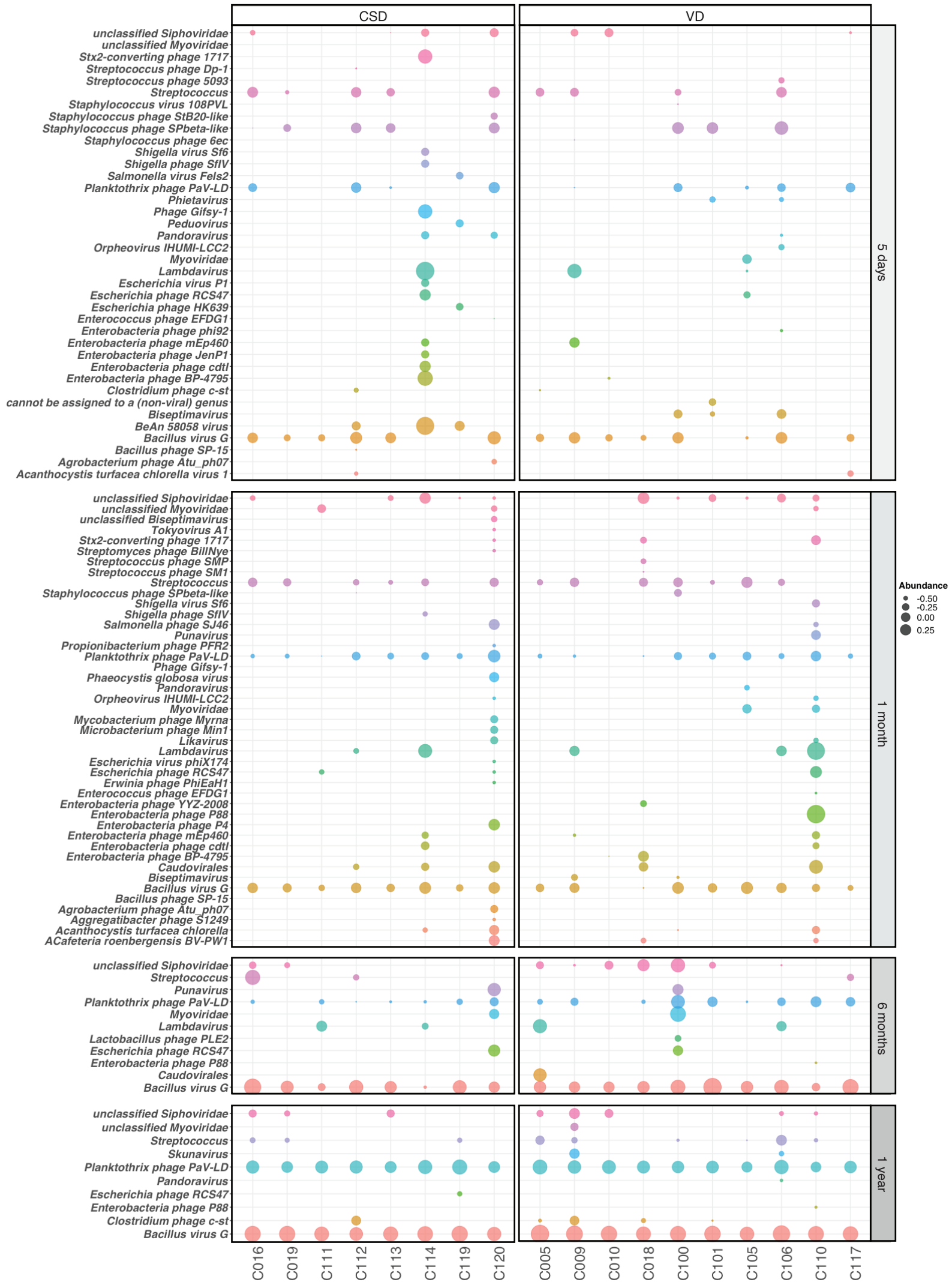

Supplement: Supplementary file 7 — Supplementary figure 6 [file 43705_2021_3_MOESM7_ESM.pdf]

Supplementary Figure. 7

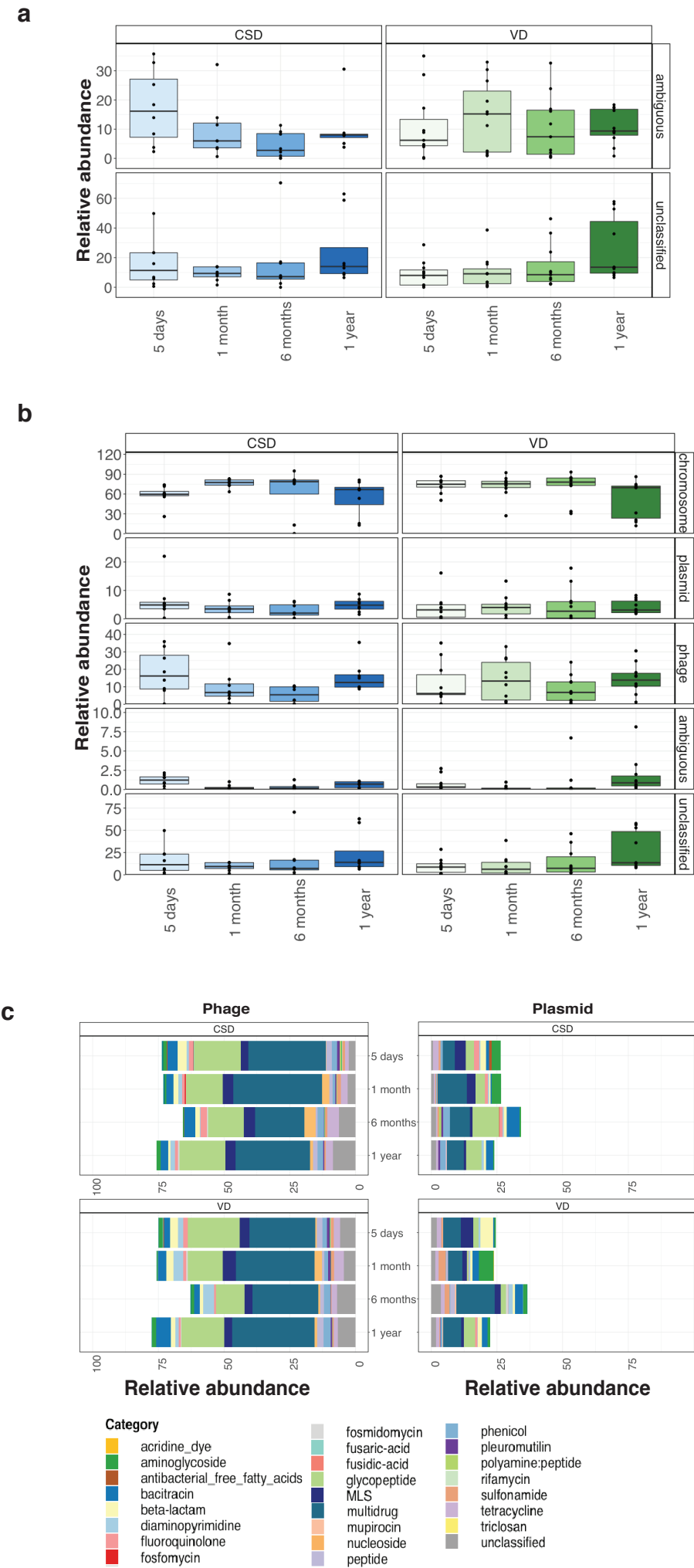

Supplement: Supplementary file 8 — Supplementary figure 7 [file 43705_2021_3_MOESM8_ESM.pdf]

Supplementary Figure. 8

**a**

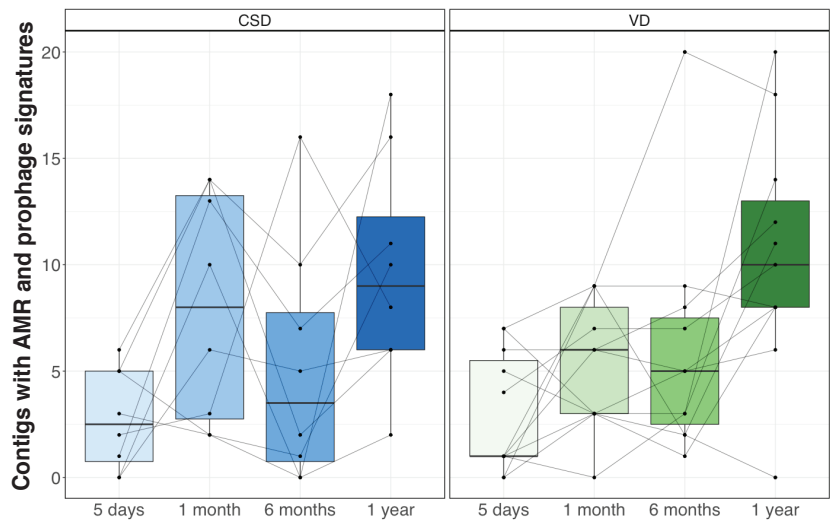

**b**

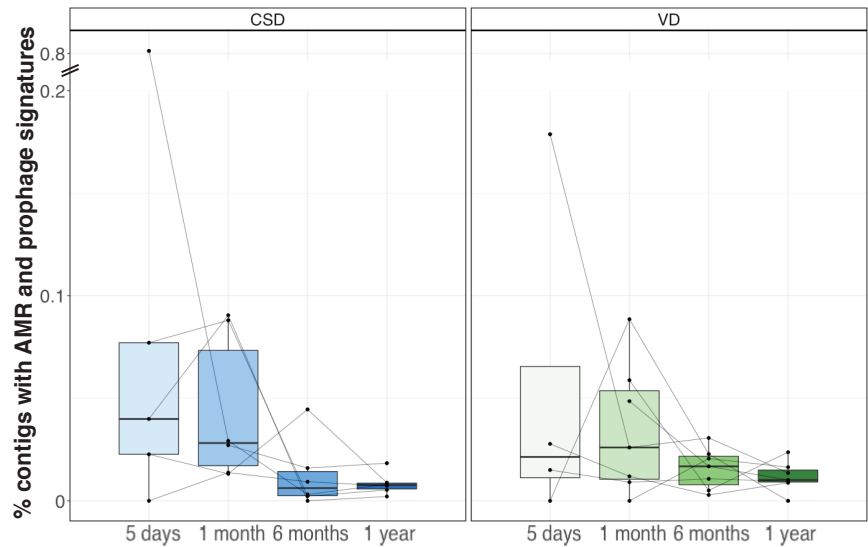

Supplement: Supplementary file 9 — Supplementary figure 8 [file 43705_2021_3_MOESM9_ESM.pdf]

**a**

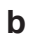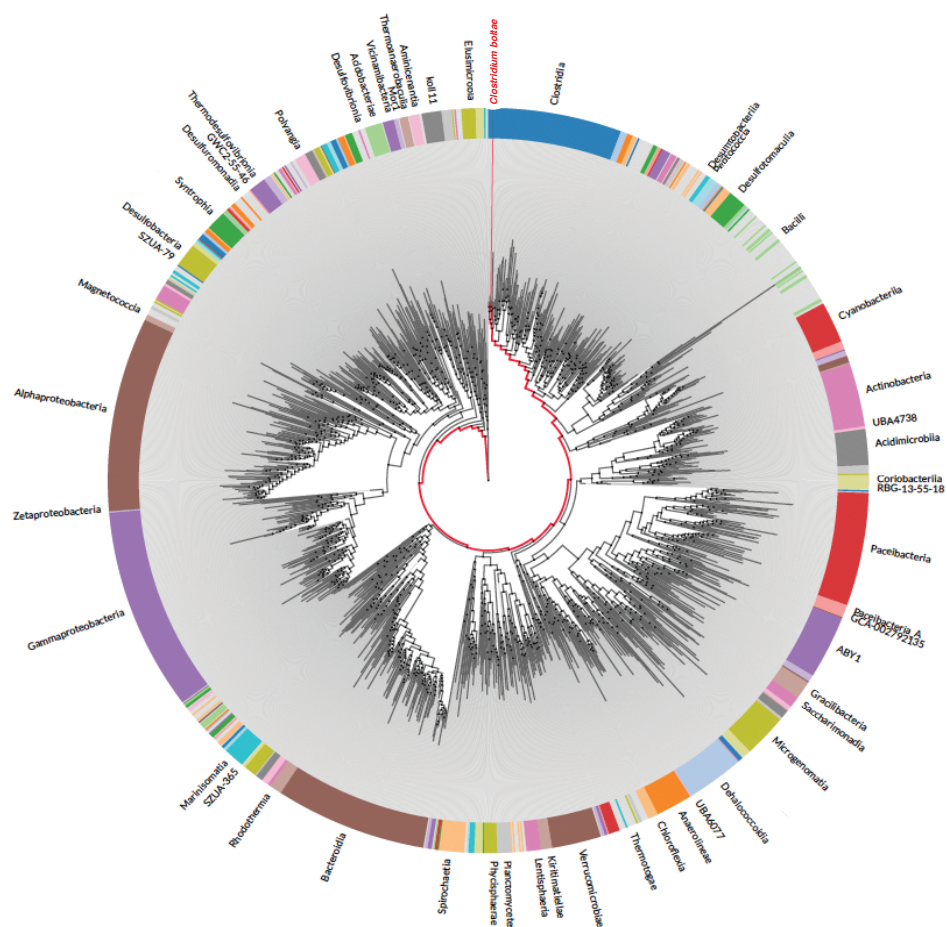

Supplement: Supplementary file 10 — Supplementary figure 9 [file 43705_2021_3_MOESM10_ESM.pdf]

Supplementary Figure. 10

**a**

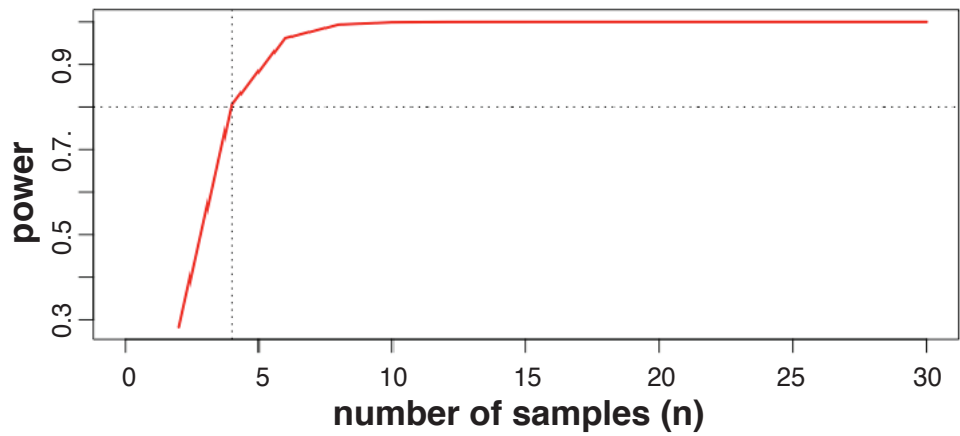

**b**

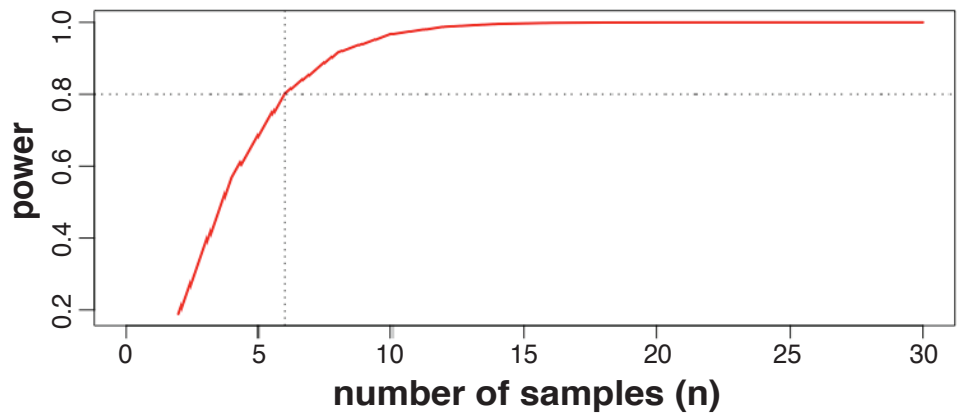

Supplement: Supplementary file 11 — Supplementary figure 10 [file 43705_2021_3_MOESM11_ESM.pdf]

**a**

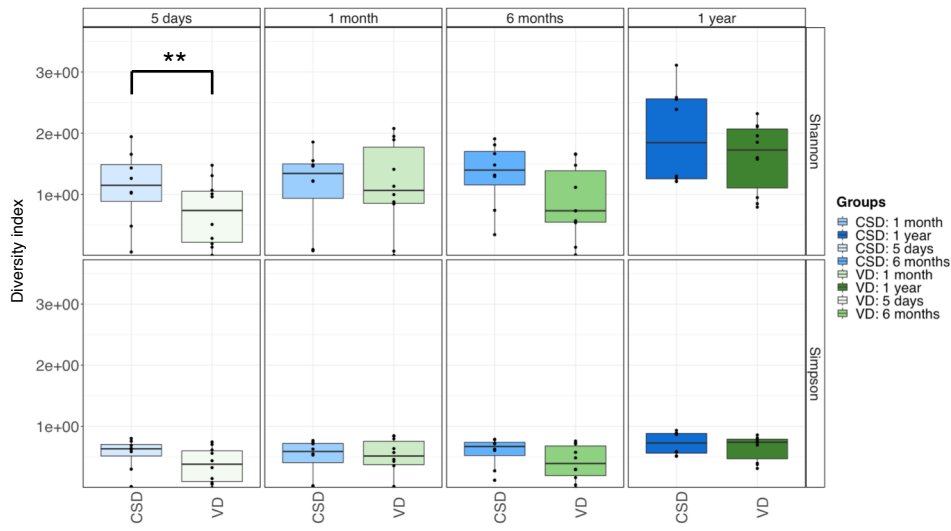

**b**

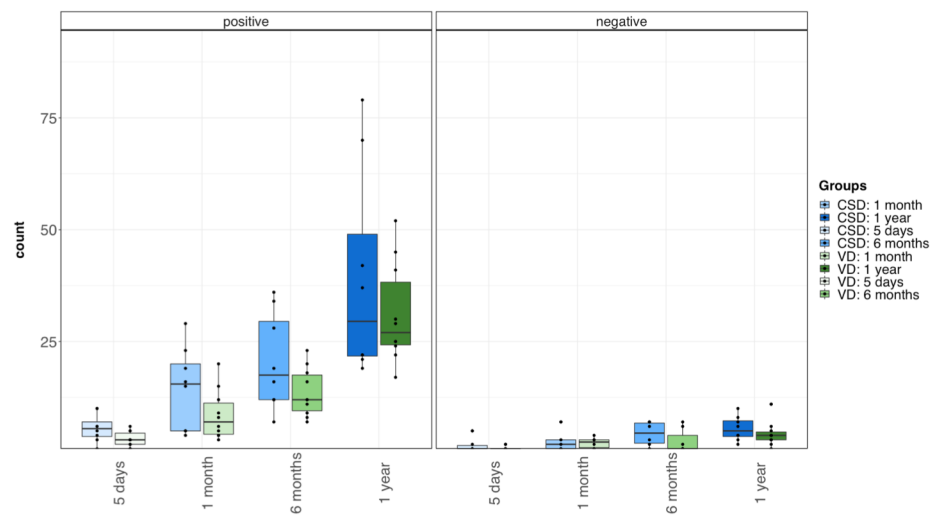

**c**

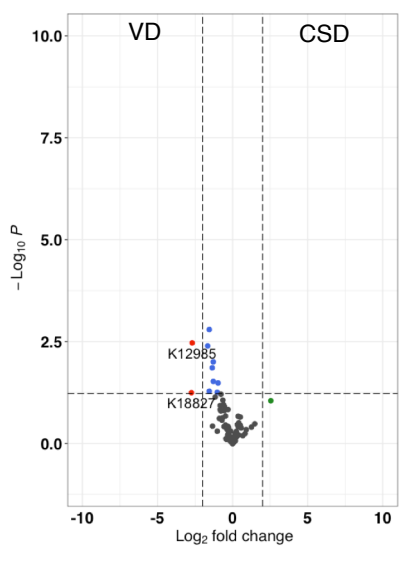

**d**

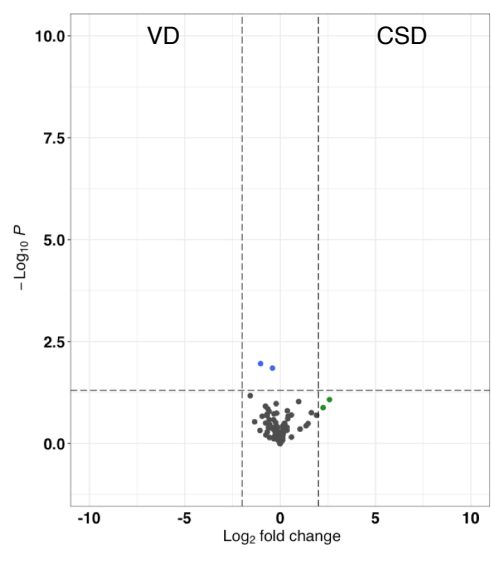

**Sig**  
● NS  
●  $\log_2$  FC  
● p-value  
● p-value and  $\log_2$  FC

Supplement: Supplementary file 12 — Supplementary figure [file 43705_2021_3_MOESM12_ESM.pdf]
